# Effectiveness of Ledipasvir-Sofosbuvir 12 Weeks After Hepatitis C Virus Genotype 1 Infection and the Factors Associated With Sustained Virologic Response: A Retrospective Study

**DOI:** 10.7759/cureus.68249

**Published:** 2024-08-30

**Authors:** Ismaeel A Alshoaibi, Abdullah Al-Gamli, Mohammed Abdullah, Basheer Abdo, Khaled H Alzanen, Mohammed Alhakamy, Mamoon Al-Namer, Faisal Ahmed, Munther Tamesh, Wadhah Mahdi, Zeyad Abdo, Marwa Mohammed

**Affiliations:** 1 Internal Medicine, School of Medicine, Ibb University, Ibb, YEM; 2 Pharmacy, University of Science and Technology, Ibb, YEM; 3 Urology, School of Medicine, Ibb University, Ibb, YEM; 4 General Practice, School of Medicine, Ibb University, Ibb, YEM

**Keywords:** yemen, ibb, sustained virologic response, hepatitis c virus, sofosbuvir, ledipasvir, interferon, non-structural protein 5b inhibitors, nonstructural protein 5a inhibitors, antiviral

## Abstract

Background: The combination of ledipasvir and sofosbuvir (LDV/SOF) has been licensed to treat genotype 1 hepatitis C virus infection (HCV) with a 12-week regimen. However, there is scant data from Yemen regarding this combination regimen. Here, we investigate sustained virologic responses (SVR) 12 weeks after HCV treatment with LDV/SOF regimens and the factors that contribute to SVR failure.

Material and Method: A retrospective cross-sectional study was conducted at Althora General Hospital in Ibb, Yemen, from June 1, 2019, to October 31, 2022, on 53 cases with HCV genotype 1 infection who received combined therapy of LDV/SOF and completed treatment for 12 weeks. The clinical characteristics and treatment follow-up were obtained from patient medical records. Factors associated with SVR failure were investigated in univariate analysis with odds ratio (OR) and 95% confidence interval (CI).

Result: The mean age was 50 ± 15.3 years, and most cases were female (n=36, 67.9%). Comorbidities were diabetes, hypertension, and fatty liver, which were represented in 12 (22.6%), nine (17.0%), and eight (15.1%) cases, respectively. A total of 13 (24.5%) patients had compensated liver cirrhosis, while the remaining 40 patients (75.5%) were non-cirrhotic healthy individuals. The baseline viral load (HCV RNA) was more than 800000 IU/mL in 21 patients (39.6%). Early virological response (ERV) was achieved in 51 patients (96.2%). After treatment, 46 of the patients (86.8%) achieved SVR at Week 12, while failure occurred in two patients (3.8%) and relapse occurred in five patients (9.4%). Blood liver enzymes, including alanine aminotransferase, aspartate aminotransferase, and alkaline phosphatase, returned to normal, with statistically significant improvements in non-cirrhotic healthy persons than compensated liver cirrhosis individuals (p= 0.006, 0.006, and 0.010; respectively). Factors associated with SVR failure were older age (OR:1.13; 95% CI: 1.03-1.30, p=0.009), presence of liver cirrhosis (OR: 5.48; 95% CI: 1.04-28.98, p=0.031), having diabetes (OR: 6.33; 95% CI: 1.19-37.93, p= 0.019), baseline higher viral load (OR: 2.27; 95% CI: 0.45-12.73, p<0.001), and not achieving EVR (OR:7.63; 95% CI: 3.77- 17.78, p= 0.009).

Conclusion: In this study, we found that LDV/SOF regimens are effective against HCV genotype one infection, allowing for the expansion of 12-week treatment for suitable patients in clinical settings. Additionally, older age, liver cirrhosis, diabetes, higher pretreatment viral load, and non-completion of EVR were associated with SVR failure. However, due to the small number of HCV genotype 1 infected individuals in this study, more corporate data is required to get a clear conclusion.

## Introduction

Hepatitis C (HCV), a viral infection, poses a global health challenge, especially in developing countries. It can be acute or chronic, and most people are unaware of infection [1]. HCV is the leading cause of liver cirrhosis and its complications, including decompensation and potentially fatal hepatocellular carcinoma (HCC) [1,2]. Chronic HCV infection affects around 50 million individuals worldwide, with 1.0 million new infections occurring each year. In 2022, 240,000 individuals died from liver cirrhosis and HCC, with 95% cure through direct-acting medications [3]. The Eastern Mediterranean region has the highest disease burden of HCV, with 12 million individuals infected [4]. In Yemen, HCV infection prevalence ranges from 0.6% to 5.1%, with the city of Ibb being one of the more affected regions with a reported rate of 1.99% [5,6]. HCV has six genotypes, with genotype 4 being the most common in Middle Eastern countries like Yemen, accounting for 63.7% of all HCV infections [7]. Developing a vaccine for HCV is challenging, and current treatments include antivirals and liver transplantation. The goal of treatment is to eradicate HCV, as measured by sustained virologic response (SVR), which is linked to lower inflammation, fibrosis, and overall mortality [7].

Interferon with ribavirin was the recommended treatment for HCV patients until 2013, with an SVR rate of 48-55% and a relapse rate of 24%. However, these treatments were associated with flu-like symptoms and depression [8]. Currently, researchers are developing direct antiviral therapy (DAT) medicines targeting HCV-specific viral proteins, potentially reducing side effects and increasing SVR rates [9]. The first HCV nucleoside polymerase inhibitor, sofosbuvir (SOF), has been approved for use with genotypes 1, 2, 3, and 4. Furthermore, a single-tablet regimen combining ledipasvir and sofosbuvir (LDV/SOF) was approved in 2014, resulting in increased SVR rates of up to 90-95% in HCV patients [9]. Terrault et al. found that LDV/SOF regimens are highly effective for HCV genotype 1 patients, justifying an eight-week treatment for eligible individuals [2].

Data on direct-acting antiviral agents (DAAs) in developing countries in Asia and the Middle East, including Yemen, is limited due to high drug costs [10]. In 2016, Gilead and Bristol-Myers Squibb granted voluntary licenses to generic firms to mass-produce cheaper generic DAAs, like LDV/SOF, with a course treatment price of $150 [11]. This strategy promises to meet the World Health Organization's (WHO) targets for HCV elimination by 2030. However, poverty and limited access to new medications make therapy clinically challenging in these countries [12].

The combination of LDV/SOF has been licensed to treat genotype 1 HCV infection with a 12-week regimen [13]. However, there are scant statistics from Yemen regarding the efficacy of this combination. Here, we investigate the SVR 12 weeks after HCV treatment with the LDV/SOF regimen in a resource-limited setting, including the factors contributing to SVR failure.

## Materials and methods

Study design and patient population

A retrospective cross-sectional study was conducted at Althora General Hospital in Ibb, Yemen, from June 1, 2019, to October 31, 2022, on 53 patients with HCV genotype 1 infection who received combined therapy of LDV/SOF (90 mg/400 mg) (Marcyrl Pharmaceutical Industries, Egypt) once daily for 12 weeks and have a verified SVR12 status.

Inclusion and exclusion criteria

The trial included adult patients with confirmed PCR HCV genotype 1 infection having either normal or compensated Child-Pugh-Turcotte score A and who received 12 weeks of LDV/SOF therapy. Exclusion criteria were age under 20, history of organ transplantation, decompensated liver disease (CTP score B or C), major comorbidities such as renal failure, cardiac failure, and advanced malignancy, and those with previous treatment failure or relapse.

Baseline collected data

Baseline data were collected from the patient's files and included age, gender, comorbidities (hypertension, diabetes, and fatty liver), liver function tests including alanine aminotransferase (ALT), aspartate aminotransferase (AST), alkaline phosphatase (ALP), total bilirubin (TB), and baseline HCV ribonucleic acid (RNA).

Liver disease severity

An expert radiologist conducted an abdominal ultrasound to evaluate liver status, spleen size, portal vein diameter, and ascites. All patients underwent an upper GI endoscopy (using the PENTAX-EPK-5000 [Pentax Corp., Tokyo, Japan]) to assess esophageal varices, grading, and gastropathy. HCV RNA was quantified using polymerase chain reaction (PCR) (using the Cobas TaqMan 48 [Roche Diagnostics, Rotkreuz, Switzerland]), with a limit of detection of 18 copies/mL [14,15].

Treatment effectiveness and safety

From HCV RNA PCR results, HCV RNA negativity was assessed 12 weeks after the end of therapy in all LDV/SOF combination patients with a valid sustained SVR 12 status. Patients who were lost to follow-up without having their HCV RNA evaluated 12 weeks after the completion of therapy were deemed non-virological failures. In contrast, those with detectable viral load at this time were considered virological failures.

Data were gathered on the treatment course, incidence of adverse events (AEs) with an assessment of their severity, and deaths with an evaluation of their relationship to antiviral therapy during and up to 12 weeks after completion.

Definitions

We defined the SVR as undetectable HCV RNA (<12 IU/mL) after 12 weeks of therapy. Treatment failure was described as having a detectable viral load 12 weeks after discontinuing DAA. Early virological response (EVR) was defined as a ≥2 log10 decline from baseline in HCV RNA or as a negative HCV RNA (<50 IU/ml) at Week 12. Patient relapse was defined as the reappearance of HCV RNA during the follow-up period in patients who had obtained negative HCV RNA after treatment [16].

Primary and secondary outcomes

The main outcome was the SVR rate at the end of treatment and the factors associated with SVR failure at 12 weeks. The secondary outcome was to compare the cirrhotic and noncirrhotic groups.

Statistical analysis

The data were analyzed using SPSS software version 21 (IBM Corp., Armonk, New York). We conducted a descriptive analysis of the entire sample, reporting quantitative data as mean ± standard deviation and qualitative variables as frequencies and percentages. The ANOVA test was used to compare quantitative variables, whereas the chi-square test or Fisher exact test was used to compare qualitative variables. The odds ratios (OR) and 95% confidence intervals (CI) were calculated to assess the strength of the associations. Statistical significance was set at p < 0.05.

## Results

The mean age was 50 ± 15.3 years, ranging between 23 years and 75 years; most patients were female (n=36, 67.9%) and above the age of 40 (n=33, 62.3%). Comorbidities were diabetes, hypertension, and fatty liver, which were represented in 12 (22.6%), nine (17.0%), and eight (15.1%) of cases, respectively. There were 13 (24.5%) patients who had compensated liver cirrhosis, and the remainder (n= 40, 75.5%) were non-cirrhotic healthy individuals. Patients with compensated liver cirrhosis were older than non-cirrhotic healthy individuals (61.7 ± 6.9 years vs. 46.2 ± 15.4 years) and were statistically significant (p=0.001). Additionally, baseline liver enzymes, including AST, ALT, and ALP, were higher in patients with compensated liver cirrhosis than in non-cirrhotic healthy individuals and were statistically significant (p= 0.023, 0.007, and 0.012, respectively). The baseline viral load (HCV RNA) was over 800,000 IU/mL in 21 patients (39.6%). The gender, comorbidities such as diabetes, hypertension, fatty liver, and baseline viral load were not statistically significant between non-cirrhotic healthy individuals and compensated liver cirrhosis. The baseline demographic and clinical characteristics of patients and among cirrhotic and non-cirrhotic groups are mentioned in Table 1.

**Table 1:** Baseline demographic and clinical characteristics of patients and among cirrhotic, and non-cirrhotic groups. Abbreviations: ALT, alanine aminotransferase; AST, aspartate aminotransferase; ALP, alkaline phosphatase; TB, total bilirubin; HCV, hepatitis C virus; SD, standard deviation; RNA, Ribonucleic acid. Note: ^a^ Data was presented as Mean± SD, while ^b^ Data was presented as n (%). P-values of < 0.05 were considered significant.

Variables	Subgroups	Total (N=53)	Cirrhosis (N= 13, 24.5%)	Noncirrhotic (N= 40; 75.5%)	p-value
Age (year)^a^	Mean ± SD	50.0 ± 15.3	61.7 ± 6.9	46.2 ± 15.4	0.001
Gender^b^	Male	17 (32.1)	6 (46.2)	11 (27.5)	0.363
	Female	36 (67.9)	7 (53.8)	29 (72.5)	
Hypertension^b^	No	44 (83.0)	9 (69.2)	35 (87.5)	0.272
	Yes	9 (17.0)	4 (30.8)	5 (12.5)	
Diabetes^b^	No	41 (77.4)	8 (61.5)	33 (82.5)	0.235
	Yes	12 (22.6)	5 (38.5)	7 (17.5)	
Fatty liver^b^	No	45 (84.9)	13 (100.0)	32 (80.0)	0.192
	Yes	8 (15.1)	0 (0.0)	8 (20.0)	
Pretreatment ALT (U/L)^a^	Mean ± SD	39.6 ± 18.9	51.6 ± 21.2	35.6 ± 16.6	0.007
Pretreatment AST (U/L)^a^	Mean ± SD	37.1 ± 21.9	48.9 ± 27.1	33.2 ± 18.7	0.023
Pretreatment TB (mg/dL)^a^	Mean ± SD	0.8 ± 0.2	0.9 ± 0.2	0.8 ± 0.2	0.140
Pretreatment ALP (IU/L)^a^	Mean ± SD	3.6 ± 0.4	3.3 ± 0.4	3.6 ± 0.4	0.012
Pretreatment Viral load (HCV RNA, log10 IU/mL)^a^	Mean ± SD	9912154.4 ± 37597461.8	14171118.5 ± 36691688.8	8527991.1 ± 38242745.0	0.643
Pretreatment Viral load (HCV RNA, log10 IU/mL)^b^	<800000	32 (60.4)	10 (76.9)	22 (55.0)	0.281
	>800000	21 (39.6)	3 (23.1)	18 (45.0)	

All patients tolerated the medication well, with no significant side effects or treatment discontinuation. The most prevalent adverse effect seen in the study group was weariness, which was reported by 41 patients (77.4%) during therapy, followed by nausea in 12 patients (22.6%), diarrhea in seven (13.2%), and headache in five (9.4%); there was no statistical significance between non-cirrhotic healthy individuals and those with compensated liver cirrhosis. No patient reported significant or dangerous adverse reactions or ceased treatment due to side effects. EVR was achieved in 51 patients (96.2%). After treatment, 46 of the patients (86.8%) achieved SVR at Week 12, while failure occurred in two patients (3.8%) and relapse occurred in five patients (9.4%). The SVR was higher in non-cirrhotic healthy individuals than in patients with compensated liver cirrhosis (37.0 (92.5%) vs. 9.0 (69.2%))and was statistically significant (p=0.031). At the end of treatment, liver enzymes including ALT, AST, TB, and ALP were higher in patients with compensated liver cirrhosis than in non-cirrhotic healthy individuals and were statistically significant (p= 0.012, 0.002, 0.048, and 0.015, respectively) (Table 2).

**Table 2:** Posttreatment characteristics and response to the treatment in total and among cirrhotic, and non-cirrhotic groups. Abbreviations: ALT, alanine aminotransferase; AST, aspartate aminotransferase; ALP, alkaline phosphatase; TB, total bilirubin; EVR, early virological response; SVR, sustained virological response; SD, standard deviation. Note: ^a^ data is presented as mean± SD, while ^b^ data is presented as n (%). P-values of < 0.05 were considered significant.

Variables	Subgroups	Total	Cirrhosis (N= 13, 24.5%)	Non-cirrhotic (N= 40; 75.5%)	p-value
Posttreatment ALT (U/L)^a^	Mean ±SD	35.7 ±19.5	47.4 ±22.6	31.9 ±17.0	0.012
Posttreatment AST (U/L)^a^	Mean ±SD	33.8 ±21.0	48.9 ±26.1	29.0 ±16.7	0.002
Posttreatment TB (mg/dL)^a^	Mean ±SD	0.8 ±0.2	0.9 ±0.1	0.7 ±0.2	0.048
Posttreatment ALP (IU/L)^a^	Mean ±SD	3.6 ±0.4	3.4 ±0.4	3.7 ±0.4	0.015
SVR^b^	Target not detected	46 (86.8)	9 (69.2)	37 (92.5)	0.095
	Relapse	5 (9.4)	3 (23.1)	2 (5.0)	
	Failure	2 (3.8)	1 (7.7)	1 (2.5)	
EVR^b^	Yes	51 (96.2)	12 (92.3)	39 (97.5)	0.987
	No	2 (3.8)	1 (7.7)	1 (2.5)	

**Figure 1:**
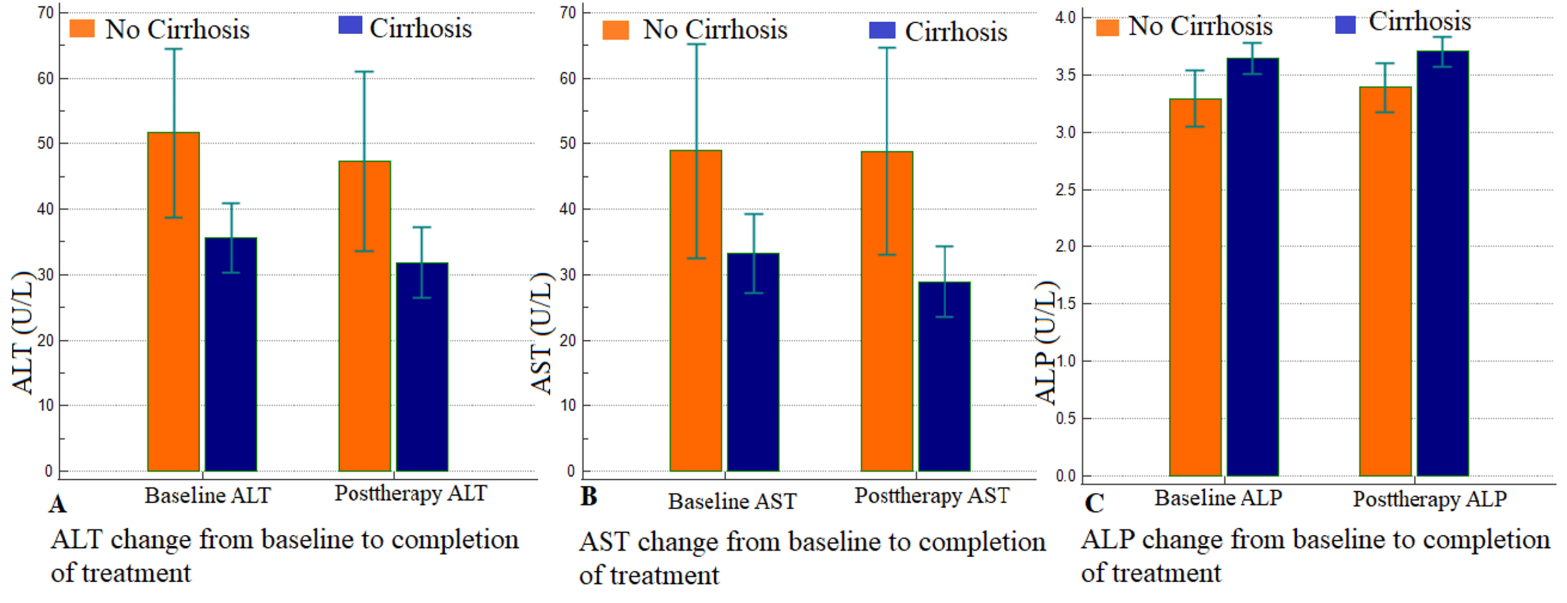
Showing the change of serum liver enzymes of ALT (A), AST (B), and ALP (C) between non-cirrhotic healthy individuals (blue box) and compensated liver cirrhosis (Orang box) from baseline to completion of treatment that were statistically significant (p= 0.006, 0.006, 0.010, respectively). Abbreviations: ALT, alanine aminotransferase; AST, aspartate aminotransferase; ALP, alkaline phosphatase. The data is represented as mean±SD.

Additionally, serum liver enzymes, including ALT, AST, and ALP, showed gradual normalization until the end of treatment and were statistically significant among non-cirrhotic healthy individuals than compensated liver cirrhosis (p= 0.006, 0.006, 0.010, respectively) (Figure 1).

Factors associated with failure of SVR

Older age (OR:1.13; 95% CI: 1.03-1.30, p=0.009), presence of liver cirrhosis (OR: 5.48; 95% CI: 1.04-28.98, p=0.031), having diabetes (OR: 6.33; 95% CI: 1.19-37.93, p= 0.019), baseline higher viral load (HCV RNA) (OR: 2.27; 95% CI: 0.45-12.73, p<0.001), and non- achieved EVR (OR:7.63; 95% CI: 3.77- 17.78, p= 0.009) were associated with failure of SVR and were statistically significant in univariate analysis (Table 3).

**Table 3:** Factors associated with failure of SVR. Abbreviations: ALT, alanine aminotransferase; AST, aspartate aminotransferase; ALP, alkaline phosphatase; TB, total bilirubin; HCV, hepatitis C virus; EVR, early virological response; SVR, sustained virological response; SD, standard deviation; RNA, ribonucleic acid; RF, reference group. Note: ^a^ Data is presented as mean± SD, while ^b^ data is presented as n (%). P-values of < 0.05 were considered significant.

Independent variable	Subgroups	SVR achieved (N=46)	SVR not achieved (N=7)	OR (95% CI)	p-value
Age^a^	Mean ±SD	47.9 ±15.2	63.7 ±6.7	1.13 (1.03-1.30)	0.009
Pretreatment ALT (IU/L)^a^	Mean (SD)	39.5 (19.8)	40.1 (12.2)	1.00 (0.95-1.04)	0.931
Pretreatment AST (IU/L)^a^	Mean (SD)	37.0 (22.3)	37.9 (20.7)	1.00 (0.96-1.04)	0.920
Pretreatment TB (mg/dL)^a^	Mean (SD)	0.8 (0.2)	0.8 (0.2)	6.84 (0.04-3347.26)	0.498
Pretreatment ALP (IU/L)^a^	Mean (SD)	3.6 (0.4)	3.4 (0.5)	0.31 (0.04-1.88)	0.205
Gender^b^	Male	16 (34.8)	1 (14.3)	RF	0.517
	Female	30 (65.2)	6 (85.7)	3.20 (0.49-63.22)	
Liver cirrhosis^b^	Noncirrhotic	37 (80.4)	3 (42.9)	RF	0.031
	Cirrhosis	9 (19.6)	4 (57.1)	5.48 (1.04-28.98)	
Hypertension^b^	No	39 (84.8)	5 (71.4)	RF	0.737
	Yes	7 (15.2)	2 (28.6)	2.23 (0.28-12.90)	
Diabetes ^b^	No	38 (82.6)	3 (42.9)	RF	0.019
	Yes	8 (17.4)	4 (57.1)	6.33 (1.19-37.93)	
Fatty liver^b^	No	38 (82.6)	7 (100.0)	RF	0.528
	Yes	8 (17.4)	0 (0.0)	1.03 (0.28-2.90)	
Pretreatment Viral load (HCV RNA, log10 IU/mL)^a^	Mean ±SD	2340003.5± 4963738.0	59672003.7±93476406.8	2.27(0.45-12.73)	<0.001
EVR^b^	Yes	46 (100.0)	5 (71.4)	RF	0.009
	Not	0 (0.0)	2 (28.6)	7.63(-3.77- 17.78)	

## Discussion

In this study, we retrospectively investigated the outcome of patients with HCV genotype 1 infection who received combined therapy of LDV/SOF and completed treatment for 12 weeks. Our results showed that 86.8% of patients achieved SVR at Week 12, while failure occurred in two patients (3.8%) and relapse occurred in five patients (9.4%). Additionally, older age, liver cirrhosis, diabetes, higher pretreatment viral load, and incomplete EVR were associated with SVR failure.

LDV is a potent inhibitor of HCV nonstructural protein 5A (NS5A), a phosphoprotein involved in viral replication, assembly, and secretion. SOF is a nucleotide analog inhibitor of HCV nonstructural protein 5B (NS5B) polymerase, the enzyme responsible for HCV RNA replication [17,18]. The FDA approved this combination in 2014, which implies a significant advancement in HCV treatment. This safe and easy regimen resulted in a high rate of SVR in genotype 1 patients [18]. Recent studies found a higher rate of SVR12 (95%) in treatment-naive HCV individuals and 94% in treatment-experienced patients [9,19]. However, it is still undetermined whether these high rates can be achieved in real-world contexts [2]. We reported high 12-week SVR rates of 86.8% among patients receiving LDV/SOF, consistent with previous studies conducted in developing nations. For example, Lacombe et al. found that SOF-based HCV treatment in Central and West Africa was feasible, safe, efficacious, and potentially scale-up with an SVR12 reaching 89% of patients [20]. In another study conducted in Yemen, Kassim et al. investigated the response of HCV patients to LDV/SOF in combination with Ribavirin among 65 patients. They found that the 12-week SVR rate was 90.8% [21]. In Rwanda, Umutesi et al. reported that the 12-week SVR rate was 87% with LDV/SOF combinations [22]. However, our SVR rate was slightly lower than other reports, such as Terrault et al., who reported SVR12s in 97% of patients receiving LDV/SOF for 12 weeks [2]. Furthermore, the 12-week SVR rate in the Afdhal et al. study was 97% [23]. The low SVR12 in our study may be due to the low sample size and monocentric design study. Additionally, our study included many cirrhotic patients (24.5%), which could have influenced the proportion of SVR achieved. Future prospective studies with more significant numbers and involving multiple centers are necessary to confirm our results.

Approximately 10-15% of HCV genotype 1 patients treated with SMV/SOF with or without RBV develop virological relapse, with cirrhosis and decompensated liver disease having greater failure rates [24]. Treatment failure is frequently associated with resistance to SMV and cross-resistance to other HCV NS3 protease inhibitors, such as grazoprevir [24]. Due to a lack of data, the appropriate retreatment protocol for this patient category is still doubtful. In this study, failure occurred in two patients (3.8%), and relapse occurred in five patients (9.4%). In another article, Osinusi et al. reported a relapse rate of HCV genotype 1 infection following LDV/SOF therapy in seven participants (28%) in the weight-based group and 10 (40%) in the low-dose group [25]. The number of reinfections varies according to HCV genotype, patient demographics, treatment era, immune system clearance, and prescribed treatment regimen.

In this study, liver cirrhosis was significantly associated with failure of SVR (OR: 5.48). The link between advanced liver disease and SVR failure was comparable to those found with direct-acting antiviral agent interferon-based regimens [2,13,25-28]. The decreased rates of SVR in cirrhotic patients may indicate that cirrhosis plays a hidden role in determining therapy response [26]. Liver cirrhosis patients often require therapy discontinuation and dosage reductions, while successful interferon treatment accelerates the urea cycle and increases ureagenesis capacity, potentially impacting their condition. For that, cirrhotic individuals should be checked for esophageal varices, HCC, and symptoms of hepatic decompensation such as hepatic encephalopathy, ascites, and infectious diseases. In general, individuals with compensated cirrhosis (CPT A) have the same likelihood of obtaining SVR with interferon regimens as non-cirrhotic patients; nevertheless, there is still a risk of decompensation and acute-on-chronic liver failure during and after therapy [9,29]. Future research is needed to investigate the efficacy of sofosbuvir and ribavirin regimens in individuals with liver cirrhosis.

Patients with chronic HCV infections have higher type 2 diabetes mellitus (T2DM) prevalence and cardiovascular risk due to the development of insulin resistance. However, SVR achieved during interferon treatment reduced T2DM incidence and prevalence [30,31]. In this study, diabetes was significantly associated with failure of SVR in univariate analysis. The link between diabetes and SVR failure was comparable to previous reports such as Arase et al., Romero-Gómez et al., Pabjan et al., and Awadh et al. [32-35]. Arase et al. found a cumulative prevalence of T2DM at varying rates, with factors such as advanced liver disease, failure to achieve SVR, baseline prediabetes, and age over 50 [32]. Romero-Gómez et al. found that insulin resistance and fibrosis stage were independent risk factors for impaired fasting glucose and T2DM in patients with chronic HCV infection [33].

The use of interferon-free direct-acting antiviral medications has reduced SVR differences between elderly and younger individuals, which could be attributed to decreasing tolerability and adherence with advanced age, as well as hepatocyte senescence, which blunts the interferon response pathway in older patients [36]. In this study, older age was significantly associated with failure of SVR. The impact of age in predicting SVR is debatable. However, most studies suggest that SVR rates were lower among elderly patients infected with HCV genotype 1 who was treated with interferon-based therapy [28,37-40]. Low SVR rates in senior individuals may owing to increased virologic nonresponse to dual therapy, the presence of side effects such as hemolytic anemia, and more concomitant comorbidities [36].

Despite higher chances of 12 weeks of SVR achievement in adult women due to estrogen section that enhances the efficacy of interferon therapy [13,16], menopause in HCV genotype 1-infected women increases liver fibrosis and reduces the response to treatment [41]. In this study, female gender was associated with SVR failure (OR: 3.20) but was not significantly significant (p=0.517), which may be due to the low sample size. Similarly, Bichoupan et al. found female gender was associated with SVR failure in univariate analysis (OR:1.96) among 215 patients treated with sofosbuvir/ribavirin but was not significantly significant in multivariate analysis [42]. In contrast, Brzdęk et al. found that male gender was identified as an independent negative predictor of therapeutic success [43].

Several studies have shown an inverse connection between pretreatment viral load and SVR [16,28,31,41]. Our findings support the hypothesis that a greater baseline viral load is significantly related to SVR failure. Additionally, in this study, incomplete EVR was significantly associated with the failure of SVR in univariate analysis. Our result was similar to other reports [28,40,41,44]. Viral kinetics accurately predict SVR, especially when viral load is undetectable at Week 4 of treatment combination [45]. In a previous review, Cavalcante et al. mentioned that rapid virological response was associated with a notably higher rate of SVR [41]. Hence, HCV RNA load should be evaluated during therapy, and patients without early or rapid virological response should be treated with innovative therapeutic techniques to reverse interferon resistance and boost treatment efficacy.

Our study found that after therapy, blood liver enzymes, including ALT, AST, and ALP, returned to normal, with significant improvements in non-cirrhotic healthy persons compared to cirrhotic individuals. Our result was similar to Villela-Nogueira et al., who mentioned that normal liver enzyme levels were associated with improved SVR rates in HCV patients without cirrhosis [31]. Low liver enzyme levels significantly predicted SVR in genotype 1 HCV patients treated with pegylated interferon and ribavirin for 48 weeks [31]. The specific source of this feature is unknown. However, it has no connection to steatosis, obesity, diabetes, alcoholism, progressive fibrosis, or oxidative stress [31].

Study limitations

The study has significant drawbacks. The study's retrospective design and limited sample size limit its ability to control for numerous confounding factors adequately. Furthermore, selection bias is probable because the study was conducted at a single academic hospital, as this may not reflect what happens in other health centers. The fundamental limitation is the reliance on secondary data, the quality of which might vary due to differences in documentation, data integrity, and record-keeping standards. The study did not consider other parameters, such as BMI and serum lipid profile.

Furthermore, laboratory test changes during treatment and economic implications for patients were noted. This omission may result in an insufficient understanding of the factors impacting treatment outcomes. A large, long-term multicenter study should be carried out to confirm our findings. Nonetheless, by providing information on potential prognostic markers for SVR 12-week failure among HCV patients, our findings add significantly to the literature on the efficacy of the LDV/SOF combination in HCV patients in resource-limited settings.

## Conclusions

This study found that LDV/SOF regimens are effective against HCV genotype one infection, allowing for the expansion of 12-week treatment for suitable patients in clinical settings. Additionally, older age, liver cirrhosis, diabetes, higher pretreatment viral load, and the non-completion of virological response were associated with SVR failure. Combination therapy of LDV/SOF regimens is an effective therapy for HCV genotype 1, which in turn prompts us to do the genotyping for all HCV-infected patients. However, due to the small number of HCV genotype 1 infected individuals in this study, more data is required to get a clear conclusion.

## References

[1] Negro F (2020). Natural history of hepatic and extrahepatic hepatitis C virus diseases and impact of interferon-free HCV therapy. Cold Spring Harb Perspect Med.

[2] Terrault NA, Zeuzem S, Di Bisceglie AM (2016). Effectiveness of ledipasvir-sofosbuvir combination in patients with hepatitis C virus infection and factors associated with sustained virologic response. Gastroenterology.

[3] World Health Organization (2022). Global health sector strategies on, respectively, HIV, viral hepatitis and sexually transmitted infections for the period 2022-2030. Global Health Sector Strategies on, Respectively, HIV, Viral Hepatitis and Sexually Transmitted Infections for the Period 2022-2030.

[4] World Health O (2016). Global health sector strategy on viral hepatitis 2016-2021. Towards ending viral hepatitis. https://iris.who.int/handle/10665/246177.

[5] Almahbashi A (2021). Hepatitis C virus epidemiology in Yemen: systematic review. Akademik Gastroenteroloji Dergisi.

[6] Gacche RN, Kaid AM (2012). Epidemiology of viral hepatitis B and C infections in Ibb city, Yemen. Hepat Mon.

[7] Ghaderi-Zefrehi H, Gholami-Fesharaki M, Sharafi H, Sadeghi F, Alavian SM (2016). The distribution of hepatitis C virus genotypes in Middle Eastern countries: a systematic review and meta-analysis. Hepat Mon.

[8] Chen CH, Yu ML (2010). Evolution of interferon-based therapy for chronic hepatitis C. Hepat Res Treat.

[9] Lawitz E, Poordad FF, Pang PS (2014). Sofosbuvir and ledipasvir fixed-dose combination with and without ribavirin in treatment-naive and previously treated patients with genotype 1 hepatitis C virus infection (LONESTAR): an open-label, randomised, phase 2 trial. Lancet.

[10] Due OT, Thakkinstian A, Thavorncharoensap M, Sobhonslidsuk A, Wu O, Phuong NK, Chaikledkaew U (2020). Cost-utility analysis of direct-acting antivirals for treatment of chronic hepatitis C genotype 1 and 6 in Vietnam. Value Health.

[11] Hill A, Tahat L, Mohammed MK (2018). Bioequivalent pharmacokinetics for generic and originator hepatitis C direct-acting antivirals. J Virus Erad.

[12] (2016). WHO. Combating hepatitis B and C to reach elimination by 2030: advocacy brief. https://www.who.int/publications/i/item/combating-hepatitis-b-and-c-to-reach-elimination-by-2030.

[13] Zarębska-Michaluk D, Brzdęk M, Jaroszewicz J (2022). Best therapy for the easiest to treat hepatitis C virus genotype 1b-infected patients. World J Gastroenterol.

[14] Chevaliez S, Bouvier-Alias M, Rodriguez C, Soulier A, Poveda JD, Pawlotsky JM (2013). The Cobas AmpliPrep/Cobas TaqMan HCV test, version 2.0, real-time PCR assay accurately quantifies hepatitis C virus genotype 4 RNA. J Clin Microbiol.

[15] Kessler HH, Cobb BR, Wedemeyer H (2015). Evaluation of the COBAS(®) AmpliPrep/COBAS(®) TaqMan(®) HCV Test, v2.0 and comparison to assays used in routine clinical practice in an international multicenter clinical trial: The ExPECT study. J Clin Virol.

[16] Gill U, Aziz H, Gill ML (2013). Rapid virological response tailors the duration of treatment in hepatitis C virus genotype 3 patients treated with pegylated interferon alfa-2a and ribavirin in Pakistan. Int J Infect Dis.

[17] Pearlman BL (2024). Direct-acting antiviral therapy for patients with chronic hepatitis C infection and decompensated cirrhosis. Dig Dis Sci.

[18] Alqahtani S, Sulkowski M (2015). Current and evolving treatments of genotype 1 hepatitis C virus. Gastroenterol Clin North Am.

[19] Kowdley KV, Gordon SC, Reddy KR (2014). Ledipasvir and sofosbuvir for 8 or 12 weeks for chronic HCV without cirrhosis. N Engl J Med.

[20] Lacombe K, Moh R, Chazallon C (2024). Feasibility, safety, efficacy and potential scaling-up of sofosbuvir-based HCV treatment in Central and West Africa: (TAC ANRS 12311 trial). Sci Rep.

[21] Kassim A, Alathwary R, Al-hogami T (2019). Response of HCV Yemeni patients to sofosbuvir/ledipasvir in combination with ribavirin. OALib.

[22] Umutesi G, Shumbusho F, Kateera F (2019). Rwanda launches a 5-year national hepatitis C elimination plan: A landmark in sub-Saharan Africa. J Hepatol.

[23] Afdhal N, Zeuzem S, Kwo P (2014). Ledipasvir and sofosbuvir for untreated HCV genotype 1 infection. N Engl J Med.

[24] Aqel B, Leise M, Vargas HE (2018). Multicenter experience using ledipasvir/sofosbuvir ± RBV to treat HCV GT 1 relapsers after simeprevir and sofosbuvir treatment. Ann Hepatol.

[25] Osinusi A, Meissner EG, Lee YJ (2013). Sofosbuvir and ribavirin for hepatitis C genotype 1 in patients with unfavorable treatment characteristics: a randomized clinical trial. JAMA.

[26] Ahmed H, Elgebaly A, Abushouk AI, Hammad AM, Attia A, Negida A (2017). Safety and efficacy of sofosbuvir plus ledipasvir with and without ribavirin for chronic HCV genotype-1 infection: a systematic review and meta-analysis. Antivir Ther.

[27] Oh JY, Kim BS, Lee CH (2019). Daclatasvir and asunaprevir combination therapy for patients with chronic hepatitis C virus genotype 1b infection in real world. Korean J Intern Med.

[28] Nguyen Thi Thu P, Ngo Thi Quynh M, Pham Van L, Nguyen Van H, Nguyen Thanh H (2022). Determination of risk factors associated with the failure of 12 weeks of direct-acting antiviral therapy in patients with hepatitis C: a prospective study. Biomed Res Int.

[29] Gane EJ, Stedman CA, Hyland RH (2014). Efficacy of nucleotide polymerase inhibitor sofosbuvir plus the NS5A inhibitor ledipasvir or the NS5B non-nucleoside inhibitor GS-9669 against HCV genotype 1 infection. Gastroenterology.

[30] Drazilova S, Gazda J, Janicko M, Jarcuska P (2018). Chronic hepatitis C association with diabetes mellitus and cardiovascular risk in the era of DAA therapy. Can J Gastroenterol Hepatol.

[31] Moraes Coelho HS, Villela-Nogueira CA (2010). Predictors of response to chronic hepatitis C treatment. Ann Hepatol.

[32] Arase Y, Suzuki F, Suzuki Y (2009). Sustained virological response reduces incidence of onset of type 2 diabetes in chronic hepatitis C. Hepatology.

[33] Romero-Gómez M, Fernández-Rodríguez CM, Andrade RJ (2008). Effect of sustained virological response to treatment on the incidence of abnormal glucose values in chronic hepatitis C. J Hepatol.

[34] Pabjan P, Brzdęk M, Chrapek M (2022). Are there still difficult-to-treat patients with chronic hepatitis C in the era of direct-acting antivirals?. Viruses.

[35] Awadh A, Badri Z, Alansari N (2024). Effects of comorbid conditions and prescribed chronic medications on the treatment plan for chronic hepatitis C infection: A cross-sectional retrospective study. Health Sci Rep.

[36] Rheem J, Sundaram V, Saab S (2015). Antiviral therapy in elderly patients with hepatitis C virus infection. Gastroenterol Hepatol (N Y).

[37] Nishikawa H, Iguchi E, Koshikawa Y (2012). The effect of pegylated interferon-alpha2b and ribavirin combination therapy for chronic hepatitis C infection in elderly patients. BMC Res Notes.

[38] Roeder C, Jordan S, Schulze Zur Wiesch J (2014). Age-related differences in response to peginterferon alfa-2a/ribavirin in patients with chronic hepatitis C infection. World J Gastroenterol.

[39] Kim HI, Kim IH, Jeon BJ (2012). Treatment response and tolerability of pegylated interferon-α plus ribavirin combination therapy in elderly patients (≥ 65 years) with chronic hepatitis C in Korea. Hepat Mon.

[40] Fried MW, Hadziyannis SJ, Shiffman ML, Messinger D, Zeuzem S (2011). Rapid virological response is the most important predictor of sustained virological response across genotypes in patients with chronic hepatitis C virus infection. J Hepatol.

[41] Cavalcante LN, Lyra AC (2015). Predictive factors associated with hepatitis C antiviral therapy response. World J Hepatol.

[42] Bichoupan K, Tandon N, Crismale JF (2017). Real-world cure rates for hepatitis C virus treatments that include simeprevir and/or sofosbuvir are comparable to clinical trial results. World J Virol.

[43] Brzdęk M, Zarębska-Michaluk D, Rzymski P (2023). Changes in characteristics of patients with hepatitis C virus-related cirrhosis from the beginning of the interferon-free era. World J Gastroenterol.

[44] Chung HJ, Lee JW, Kim YS, Lee JI (2013). Prediction of sustained virologic response based on week 4 and week 12 response in hepatitis C virus genotype 1 patients treated with peginterferon and ribavirin: assessment in a favorable IL28B allele-prevalent area. Intervirology.

[45] Navaneethan U, Kemmer N, Neff GW (2009). Predicting the probable outcome of treatment in HCV patients. Therap Adv Gastroenterol.

